# Tertiary Trauma Survey on Emergency Department Observational Units: A Systematic Literature Review

**DOI:** 10.7759/cureus.53187

**Published:** 2024-01-29

**Authors:** Tamkeen Pervez, Mehreen Malik

**Affiliations:** 1 Emergency Medicine, Combined Military Hospital, Rawalpindi, PAK; 2 Family Medicine, Heavy Industries Taxila (HIT) Hospital, Taxila, PAK

**Keywords:** delayed diagnosis, missed injuries, adult, clinical decision unit, emergency observation units, observational medicine, major trauma, tertiary trauma survey

## Abstract

In today's competitive world with a fast-paced lifestyle, trauma is on the rise and is globally recognized as the leading cause of mortality, morbidity, and disability. Despite the development of major trauma centers and the introduction of advanced trauma training courses and management guidelines, there remains a substantial risk of missed or delayed diagnosis of injuries with potentially life-changing physical, emotional, and financial implications. The proportion of such incidents is potentially higher in busy emergency departments and developing countries with fewer dedicated major trauma centers or where focused emergency and trauma training and skills development is still in its infancy. In the last decade, tertiary trauma surveys have been recognized as an important re-assessment protocol in reducing such missed injuries or delayed diagnoses in patients involved in major trauma. This naturally leads to the presumption that tertiary trauma surveys could also play an important role in observational medicine. This also brings into question whether a standardized tertiary trauma survey of major trauma patients on emergency observation units could reduce missed injuries, especially in low-income countries with fewer resources and trauma expertise.

Thus, the purpose of this systematic literature review is to explore the potential role of tertiary trauma survey as a tool to reducing missed or delayed diagnosis in the emergency observation units and its applicability and feasibility in less-developed healthcare systems and in low- and middle-income countries.

A broad-based systematic literature review was conducted to include electronic databases, grey literature, reference lists, and bibliographies using the keywords: tertiary trauma survey, major trauma, observational medicine, emergency observation units, clinical decision unit, adult, missed injuries, and delayed diagnosis. Over 19,000 citations were identified on initial search. Following a review of abstracts, application of inclusion and exclusion criteria, and review of the full article, 19 publications were finally selected for the purpose of this systematic literature review.

Current evidence shows a general trend that tertiary trauma surveys performed 24 hours after admission play an important role in identifying injuries missed at the time of initial primary and secondary survey, and its implementation in observational medicine could prove beneficial, especially in resource-depleted healthcare systems.

## Introduction and background

The global burden of mortality, morbidity, and disability following trauma surpasses that of any other disease, and it has been declared the leading cause of death between the ages of five and 29 years [1,2]. Subsequently, trauma accounts for a significant number of emergency department (ED) presentations worldwide.

The tangible and intangible costs of the consequences of trauma are high and a burden on the international economy [2]. The last couple of decades have seen the institution of several measures to mitigate the adverse outcomes of trauma. These include introducing evidence-based guidelines and protocols, developing dedicated intense advanced trauma training courses (e.g., Advanced Trauma Life Support (ATLS)) and trauma non-technical training (TNT), creating trauma teams and trauma team leaders (TTL), and establishing major trauma centers (MTC), trauma registry and national trauma networking (NTN). Despite such substantial advancements in trauma management, the reported rate of missed injuries among trauma patients ranges between 1.5% and 40% [3]. Most of this data comes from high-income countries (HIC) with well-established major trauma centers and a robust trauma management system. Keeping in mind the trauma management disparities in healthcare and a literature deficit from the less developed overburdened healthcare systems and low- and middle-income countries (LMIC), the actual number of missed injuries might be exponentially higher [4]. Missed injuries, also referred to as delayed diagnosis of injury (DDI), are defined as injuries in trauma patients that were not recognized at the time of the primary or secondary survey and have been broadly categorized into three types by Zamboni et al., 2014 [5]. Injuries missed on the primary and secondary surveys but identified within 24 hours are categorized as type I DDI. Type II DDI refers to injuries missed on the primary, secondary, and first tertiary surveys, but identified in-hospital more than 24 hours after admission. Injuries missed at all assessments during hospital admission but identified on follow-up or re-attendance are categorized as type III DDIs. 

Observational medicine (OM) is an integral part of emergency medicine (EM), and according to Lily et al 1985, approximately 2.5% of trauma patients are admitted to the emergency department observational units (EDOU) [6,7]. The general purpose of EDOU, also known as clinical decision unit (CDU), short-stay unit (SSU), or emergency observation unit (EOU), is a short-term admission of patients for 24-hour observation and monitoring, and to ensure any essential urgent investigations or reviews [6,7]. This ensures adequate monitoring, comprehensive re-assessment, and safe discharge from the ED while avoiding unnecessary in-hospital admissions. With respect to trauma patients, an EDOU plays an important role as a vast number of trauma patients do not need formal admission but benefit from a short EDOU admission [7]. These patients form a substantial subset of poly-trauma patients with low-to-moderate risk trauma, in whom it would be prudent to perform a comprehensive re-assessment prior to discharge to identify type I missed injuries. Consequently, the proportion of type II missed injuries is likely to decrease as well. In a 2015 MTC peer review [8], a full tertiary examination of the patient in the form of a tertiary trauma survey (TTS) has been recommended as the standard of care for major trauma patients. Evidence suggests that TTS can reduce the number of missed injuries by 4% [9].

The purpose of this systematic literature review is to examine the role of TTS within 24 hours and explore its potential as a simple and effective intervention in reducing the number of DDIs in adult trauma patients admitted to the EDOU. Its feasibility and applicability are likely to have a marked impact on improving the standard of care in trauma patients, especially in underdeveloped healthcare systems and LMICs with rudimentary trauma management skillset catering to a disproportionately large group of trauma patients on the world map.

## Review

Methodology

Search Strategy

An initial search using TTS, EOU, Trauma, and OM was unproductive. To maximize the number of relevant citations, a broad-based literature search was conducted to identify articles discussing the role of TTS in EDOU on adult patients with major trauma using the keywords tertiary trauma survey, major trauma, observational medicine, emergency observation unit, clinical decision unit, adult, missed injuries, and delayed diagnosis. Electronic databases utilized for the search were MEDLINE, CINAHL, EMBASE, Google Scholar, Cochrane Library, PubMed, and Ovid. A search of the grey literature was also conducted, and the reference lists were inspected to identify relevant additional literature. In addition, the bibliographies of all relevant publications were cross-referenced.

Inclusion and Exclusion Criteria

The search included all publications involving the adult population in the English language from 1950 to October 2023. Table 1 illustrates the inclusion and exclusion criteria applied to data selection.

**Table 1:** Selection criteria for literature review CDU - clinical decision unit; EDOU - emergency department observation unit; EOU - emergency observation unit; SSU - short stay unit; TTS - tertiary trauma survey

Inclusion criteria	Exclusion criteria
English language	Trauma due to specific mechanisms, esp. major incident
Adult (age 16 years or greater)	Trauma resulting in specific isolated injury
Requiring 24-hr admission	Primary endpoint identified exclusively as a certain type of injury
Formal tertiary trauma survey was performed/ documented	Studies focusing on a single component of TTS
Major trauma and non-trauma centers	Book chapters, News articles, Conference proceedings
CDU/ EOU/ SSU/ EDOU	Opinions, comments, review letters

Quality Assessment

Quality assessment of the included studies was done using the Newcastle-Ottawa scale (NOS). 

Outcomes of Interest

The primary outcome was the incidence of type-I DDIs detected on TTS within 24 hours of initial assessment in patients presenting with polytrauma, preferably in the EDOU setting. Secondary outcomes included the impact of standardized TTS on the detection rate of DDI at TS, the association between severity of initial injury and DDI, impact of type-I DDI detection rate on type-II, and type-III missed injuries. Another outcome was the efficacy of TTS on the skill level of performing physicians and the association between early detection of missed injuries and hospital length of stay (LoS).

Results

Study Selection 

**Figure 1:**
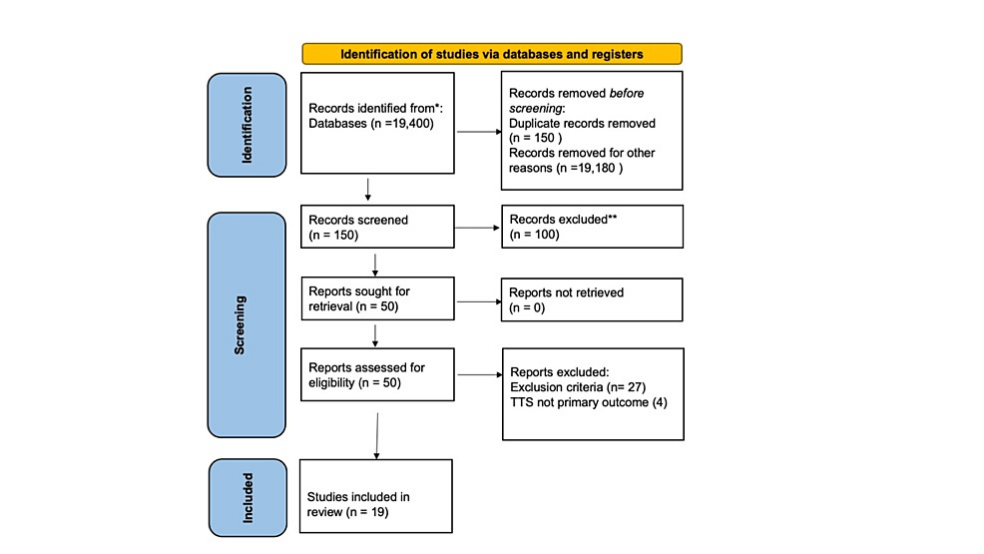
PRISMA flowchart showing study selection for the systematic literature review PRISMA - Preferred Reporting Items for Systematic Reviews and Meta-Analyses; TTS - tertiary trauma survey

Two independent reviewers diligently assessed the titles to find relevant articles. Both reviewers proceeded to scrutinize the abstract further and introduce relevant articles. Inclusion-exclusion criteria (Table 1) were applied, and reference lists were also inspected to identify additional relevant literature. Initial searches identified more than 19,000 citations. After screening for relevance, removal of duplicates, and application of eligibility criteria, 50 were selected for abstract and full article review. Following a careful examination by both reviewers, 19 publications were finally included for the purpose of this systematic literature review (Figure 1).

Study Characteristics

The selected literature included six retrospective studies, two randomized control trials (RCT), three literature reviews, one meta-analysis, and seven prospective studies [5, 10-27]. The methodology of all relevant publications included in the review was assessed individually. The baseline characteristics, outcome summary, and quality assessment of each selected study is described in Table 2 and Table 3, respectively.

**Table 2:** Baseline characteristics and outcome of the reviewed literature MIG - missed injury group; LoS - length of stay in hospital; TS - tertiary survey; TTS - tertiary trauma survey; DDI - delayed diagnosis of injury; WBCT- whole body computer tomography; SCT - selective computer tomography; RCT - randomized controlled trial; CSMI - clinically significant missed injury

Author	Country	Objective	Methodology	Results
Ju et al., 2022 [10]	Korea	To provide basic data for the development of a protocol for effective TTS to diagnose and treat missed injuries.	Design: retrospective analysis; Site: National Trauma Center; Duration: 2019-2021; Sample size: 807 patients; Time TTS: 24 hrs; Standardized TS: yes	MIG: n=73 patients; Injury sites: n=119; LoS: MIG > non-MIG; Type I: n=28 (23.5%) patients; Type II: n=90 (75.6%); Type III: n=1 (0.8%)
Underwood et al., 2022 [11]	Australia	To determine the utility of TS in patients subjected to early imaging protocol (WBCT or SCT) following trauma.	Design: retrospective analysis; Site: level-II trauma center; Duration: 2017-2019; Sample size: 507 patients; Time TTS: not specified; Standardized TS: yes	Type I CSMI: n=6 (1.18%); Type III: n=4; Mean LoS for WBCT: n=2.4 days
Suda et al., 2021 [12]	Germany	To evaluate the residual risk of missed life-threatening despite adherence to strict guidelines	Design: retrospective study; Site: level-I trauma center; Duration: 2016-2019; Sample size: 2694 patients; Time TTS: Not clear; Standardized TS: not clear	Failed to identify hollow-organ LTI: n=7 (0.26%) patients: type I LTI n=1; type II LTI n=6
Aert et al., 2020 [13]	Netherlands	The yield of TTS in patients with minor (AIS 1) or moderate (AIS 2) injuries	Design: retrospective cohort study; Site: level-II trauma center; Duration: 2015-2018; Sample size: 338; Time TTS: 24 hrs; Standardized TS: yes	Type I: n=12 patients (3.1%); Type I CSMI: n=4; Type II: n=1 patient (0.30%)
Stevens et al., 2018 [14]	USA	An overview of the literature on missed injuries in the trauma population and the role of orthopedics in TTS	Design: narrative literature review	Treating orthopedic surgeons should perform TS. TS can often pick up previously undiagnosed injuries.
Ferree et al., 2016 [15]	Netherlands	To determine the rate of DDI and factors associated with DDI in polytrauma patients	Design: retrospective cohort study; Site: level-I trauma center; Duration: 2007-2012; Sample size: 1416; Time TTS: 24 hrs; Standardized TS: yes	MIG/DDI: n=172 patients (12%); Type I: n=38 patients (22%); Type II: n=109 patients (63%); Type III: n=25 patients (15%); Of note: 2 patients had both type I & II injuries; and four patients had types I, II & III injuries.
Hajibandeh et al., 2015 [16]	UK	A systematic review of literature aimed at identifying comparative evidence about the effect of TS on missed injury rate in trauma patients.	Design: metanalysis; Duration: til June 2014; Studies: RCTs and observational studies; Sample size: 12,581 patients; Time TTS: variable; Standardized TS: variable	Pooled date to include seven studies (four prospective, three retrospective). Increased MIG rate: OR 2.65; CI 95%; p=0.003. Reduced MIG rate: OR 0.63; CI 95%; p=0.01. No effect on mortality rate: OR 0.71; CI 95%; p=0.01.
Zamboni et al., 2014 [5]	Brazil	To show the importance of TTS in trauma patients for diagnosing injuries undetected at the time of initial survey	Design: prospective observational study; Site: single-center; Duration: 2012-2013; Sample size: 526 patients; Time TTS: 24 hrs; Standardized TS: yes	Type I: n=57 injuries in 40 patients (7.6%). Of these, CSMI n=11 injuries 10 patients (19.3%)
Mirhadi et al., 2014 [17]	UK	Focus on TS in trauma patients and its impact.	Design: narrative literature review	TS is a complete summation and cataloging of a patient's injuries and is important in trauma patients.
Keijzers et al., 2014 [18]	Australia	Evaluate missed injury rates in admitted trauma patients before & after formalization of TTS.	Design: prospective cohort study with before and after design (RCT); Site: level-II TC; Duration: 2009-2010; Sample size: 487 patients (pre n=235, post n=252); Time TTS: 24 hrs or when patient regained consciousness. Standardised TS: post-group only	Type I: n=6/65 (9.2%) in pre-gp; Type I: n=10/106 (9.4%) in post-gp; Type II: n=3/170 (1.8%) in pre-gp; Type II: n=2/145 (1.4% in post-gp; Combined Type I & II: pre-intervention n=9/235 (3.8%); post-intervention n=12/252 (4.8%); Type III: n=11.5-13.7% (at one month); Type III: n=33.-3.8% (at six months)
Keijzers et al., 2012 [19]	Australia	Effect of TS on missed injuries in trauma: a systematic review	Design: systematic review; Included 10 observational studies	Type I: n=4.3%; Type II: n=1.5%; Routine TTS increases Type I and reduces Type II.
Giannakopoulos et al., 2012 [20]	Netherlands	To determine the frequency, type, and implications of missed injuries.	Design: data extracted from (REACT trial); Site: two level-I trauma centers; Duration: 2005-2007; Sample size: 1124 patients; Time TTS: 24 hrs or when patient regained consciousness; Standardized TS: yes	Missed injuries: n=122 injuries in 92 patients (8.2%); Type-I: n=26 injuries (21%); Type-II: n=72 injuries (59%)
Keijzers et al., 2011 [21]	Australia	TS performance and rate of missed injuries in a hospital without a dedicated trauma service	Design: retrospective descriptive study; Site: level-II regional trauma center without dedicated trauma services; Duration: 2008-2009; Sample size: 252 patients; Time TTS: 24 hrs; Standardised TS: yes	Type I: n=6 injuries; Type II: n=2 injuries; Combined type I & II: n=9 injuries in 8 patients (3.2%); CSMI: n=2 injuries; TS Compliance rate: n=51 (20%) polytrauma patients
Huynh et al., 2010 [22]	USA	TS within 48 hrs of admission performed by MLPs, impact on residents' workload.	Design: prospective evaluation; Site: level-I trauma center; Duration: 2007-2008; Sample size: 5143 patients; Time TTS: 48 hrs; Standardised TS: yes	TS compliance: n=3393 patients (66%); Type I &II combined: n=80 patients (1.5%); CSMI: 9% 80 patients; Residents' workload reduced by 1802 hours.
Howard et al., 2006 [23]	USA	To assess the statistical significance of missed injuries discovered through tertiary examination	Design: prospective observational study; Site: community level-II trauma center; Duration: Jul-Sept 2006; Sample size: 90 patients; Time TTS: 24 & 48 hrs; Standardised TS: yes	Total injuries: n=589 in 90 patients; Type I & II combined: n=16 injuries (3%) in 13 patients (1.5%)
Biffl et al., 2003 [24]	USA	Implementation of TS impact on the incidence of missed injuries in trauma patients admitted to ICU.	Design: prospective study, with before and after design (RCT); Site: level-I regional trauma center; Duration: 1997-2001; Sample size: 6854 (3412 pre-TS; 3442 post-TS); Time TTS: 24 hrs; Standardised TS: yes	Pre-group type I: n=39/690 patients (5.7%); Post-group type I: n=36/1048 patients (3.4%); A standardized TS reduced rate of type I missed injury rate by 36%.
Buduhan et al., 2000 [25]	Canada	To determine the incidence of missed injuries within a Canadian level-I TC.	Design: prospective; Site: level-I trauma center; Duration: 1995 -1997; Sample size: 567; Time TTS: > 24 hrs including post-discharge follow-up; Standardized TS: not clear	Total injuries: n=46/567 patients (8.1%); Type III: 11.1% of all injuries; Mortality: n=1; CSMI: n=7 patients; Potentially avoidable missed injuries: 56.3%; Unavoidable injuries: 43.8%
Janjua et al., 1998 [26]	Australia	Evaluation of missed injuries and the role of tertiary trauma survey	Design: prospective evaluation; Site: acute trauma center; Duration: 1996; Sample size: 206; Time TTS: 24 hrs; Standardised TS: not clear	Total injuries: n=798 injuries in 206 patients; Missed injuries: n=309 (39%) injuries in 134 (65%) patients' Type I: n=56% of 309 injuries; Type I CSMI: n=53/59 injuries (90%); Complications in CSMI: n=11 patients; Morality: n=2 patients
Enderson et al., 1990 [27]		TTS a prospective study of missed injuries	Design: prospective study; Duration: three months; Sample size: 399 patients; Time TTS: not clear Standardised TS:nNo	Missed injuries: n=41 injuries in 36 patients (9%); CSMI: n=21; Missed injury detection rate changed from 2% in a previous retrospective study to 9% in this study.

**Table 3:** Quality assessment of included studies: Newcastle-Ottawa scale (NOS) Quality of study: ≥7 good, 2-6 fair, ≤1 poor

Author	Selection				Comparability	Outcomes			Total score	Quality of study
	Representativeness of the exposed cohort	Selection of the non-exposed cohort	Ascertainment of exposure	Demonstration that outcome of interest was not present at the start/outset of the study	Comparability of cohorts on the basis of design analysis	Assessment of outcome	Long enough follow-up for outcomes to occur	Adequacy of follow-up of cohorts		
Ju et al., 2022 [10]	*	*	*	*	*	*	*	*	8	Good
Underwood et al., 2011 [11]	*	*	*		*	*	*	*	7	Good
Suda et al., 2021 [12]		*	*	*			*	*	5	Fair
Aert et al., 2020 [13]	*	*	*	*	**	*	*	*	9	Good
Stevens et al., 2018 [14]				*	*	*			3	Fair
Ferree et al., 2016 [15]	*	*	*	*	*	*	*	*	8	Good
Hajibandeh et al., 2015 [16]	*	*	*	*	*	*	*	*	8	Good
Zamboni et al., 2014 [5]	*	*	*	*	**	*	*	*	9	Good
Mirhadi et al., 2014 [17]				*	*	*			3	Fair
Keijzers et al., 2014 [18]	*	*	*	*	**	*	*	*	9	Good
Keijzers et al., 2012 [19]	*		*	*	**	*		*	6	Fair
Gianakopulos et al., 2012 [20]	*	*	*	*	*	*	*	*	8	Good
Keijzers et al., 2011 [21]	*	*	*	*	*	*	*	*	8	Good
Huynh et al., 2010 [22]	*	*	*	*	*	*			6	Fair
Howard et al., 2006 [23]	*	*	*	*	*	*	*	*	8	Good
Biffl et al., 2003 [24]	*	*	*	*	*	*	*	*	8	Good
Buduhan et al., 2000 [25]	*	*		*	*	*	*	*	6	Fair
Janjua et al., 1998 [26]	*	*	*	*	**	*	*	*	9	Good
Enderson et al., 1990 [27]	*	*	*	*	*	*	*	*	8	Good

All 19 citations considered for this systematic literature review, ­­­invariably emphasize the importance of TTS in reducing the number of DDI in trauma patients in various hospital environments with varying injury severity. Only one prospective RCT with a reasonable sample size has ever been done [18]. None of the studies were conducted in the EDOU, though the results of a few may be applicable to the EDOU set-up due to similar patient cohorts and timeframe for TTS [13-15, 17, 23,24, 26]. A description of the strengths and limitations of the reviewed literature and their applicability to EDOU is outlined in Table 4.

**Table 4:** Analysis of reviewed literature EDOU - emergency observation unit; QA - quality assessment; SCT selective computer tomography; TC - trauma center; TS - tertiary survey; TTS - tertiary trauma survey; WBCT - whole-body CT

Authors	Strengths	Limitations	Conclusion
Ju et al., 2022 [10]	TS within 24 hours. Data interpretation in terms of number of patients as well as number of injuries. Use of power calculations. Appropriate classification of missed injuries.	Retrospective, single-center study (generalisability). Only patients with serious injuries at the time of initial assessment were included.	Most patients in EDOU are those who suffer from moderate to mild injuries. Therefore, the results of this study are not truly representative of the EDOU patient cohort.
Underwood et al., 2022 [11]	Level-II trauma center with less advanced trauma services. Decent sample size. Standardized TS form. Use of power calculations and appropriate statistical tools.	Potentially significant number of patients from ED were not included due to poor documentation. Timing of TS is not clearly defined. Selection bias as patients referred to Level-I TC were not included.	Retrospective study of patients who underwent TS & initial radiological studies, does not include a significant proportion of patients normally detained in EDOU.
Suda et al., 2021 [12]	Clear definition of missed injuries. Significantly sized sample size. Retrieval of information from an electronic database. Use of power calculations and statistical tools. Low risk of bias.	Single-center study (generalisability). Initial assessment includes advanced radiology and trauma assessment that could reduce the number of missed injuries anyways. Timing of TS is variable. Significant number of patients lost to follow-up; those discharged from ED and returned or presented elsewhere with missed injuries are not mentioned.	Due to variations in timing of TS and initial advanced investigations, the relevance of these results with respect to adult trauma patients on EDOU is questionable.
Aert et al., 2020 [13]	Level-II trauma center with less advanced trauma services highlights the importance of TTS. Appropriate sample size. Appropriately defined types of missed injuries. Use of power calculations. Low risk of bias. TS in defined time period (24 hrs).	Single-center study.	TTS performed at 24 hours increased the rate of missed injury detection of all types & can be implemented in the EDOU.
Stevens et al., 2018 [14]	Provides an overview of the importance of TTS.	A narrative literature review from the perspective of an orthopedic surgeon with a high risk of bias by design. No pre-defined methodology.	Advocates the importance of TTS in reducing the rate of missed injuries, supporting its applicability in EDOU.
Ferree et al., 2016 [15]	Good sample size. Data accuracy & validation improved by checking all electronic records. Good follow-up rate (78%). Well-defined inclusion criteria.	Single-center study. Level-I Trauma Centre. No power calculations. Moderate risk of bias.	Strong study identifying the importance of TTS to reduce the rate of DDI. The results of this study are applicable to the EDOU set-up.
Hajibandeh et al., 2015 [16]	Low risk of bias. OR calculations. PRISMA QA tool. NOS to reduce bias	Metanalysis, including clinically diverse trials. Heterogeneity of sample populations. Variation in definitions of DDI	Due to heterogeneity of the population involved, the relevance of these results with respect to adult trauma patients on EDOU cannot be determined.
Zamboni et al., 2014 [5]	Consistency in TTS maintained by three dedicated resident physicians & use of systemized, protocol-driven clinical examination. Low risk of implicit bias.	Limited generalizability & applicability due to single-center study in Brazil. Does not include all poly-trauma patients; focuses only on those admitted with confirmed MSK injury requiring surgery.	The population of this study is not representative of the EDOU population.
Mirhadi et al., 2014 [17]	Provides a broad overview of the importance of TTS.	Narrative literature review with a high risk of bias by design. No pre-defined methodology.	Advocates the importance of TTS in reducing the rate of missed injuries, supporting its applicability in EDOU.
Keijzers et al., 2014 [18]	First of its kind. Appropriate sample size 1- & 6-month telephone follow-up post-discharge. At least five attempts made to contact patient before considering 'lost to follow-up'. State death registry utilized to identify deaths. Use of standardized TTS forms for consistency and accuracy. Use of power calculations. STROBE statement QA tool. Low risk of bias.	Single center, low generalizability. Pragmatic approach. Poor TTS compliance (27-42%). Inadequate follow-up (50% at one month; 40% at six months). Type III injury reliability and accuracy dependent on self-presentation. Significant number of patients in the pre-test group also underwent TTS.	Poor compliance to TTS implementation; a significant number of control group undergoing TTS fail to reflect truly on the effects of TTS & therefore does not truly represent the EDOU set-up.
Keijzers et al., 2012 [19]	Extensive database search. No language restriction. 10 observational studies included. PRISMA standard QA tool. NOS to reduce bias.	Observational studies only. No randomized studies. 1 of 10 studies had a different primary endpoint. Geographical Heterogeneity. Includes 1 study targeting pediatric population. Definition of missed injury variable in different studies.	Due to vast heterogeneity in sample groups, results of this systematic analysis are applicable to the role of TTS in the EDOU.
Giannakopoulos et al., 2012 [20]	Multi-center study. Large cohort. Power calculations. Good follow-up rate. Good documentation.	Both trauma centers are in the same geographical location, and therefore, the generalizability of results is questionable. Sample group is the same as the REACT-trial group. Missed injury detection rate because of TTS is a surrogate endpoint. As this study is part of the REACT trial, with patients undergoing radiological investigation was far more than normal, there could be an increased injury detection rate. TTS was not documented as a structured note. Moderate risk of bias.	This study does not differentiate injuries that were detected exclusively because of formalized TTS. Being part of the REACT trial, it can be assumed that the amount & quality of radiological investigations was above average. Therefore, the results of this study are not applicable to the EDOU set-up.
Keijzers et al., 2011 [21]	Lack of a dedicated trauma team emphasizes the importance of missed injuries at TTS in moderate to low-risk groups with less trauma management expertise.	Single-center study with questionable generalizability. No structured TTS documentation. TTS performed by physicians at the discretion of the admitting team. TTS performance rate very low (20%). No follow-up after discharge to identify Type III missed injuries. Moderate risk of bias.	Due to poorly documented informal TTS, the results of this study may not be a true representation of the missed injuries.
Huynh et al., 2010 [22]	Good sample size. Structured TTS Dedicated MLPs to perform TTS.	Single-center study, limiting generalizability. TTS performed at 48 hrs. 12% of the entire cohort did not undergo TTS due to manpower shortage. Restricted to penetrating injuries only. Moderate risk of bias.	The results of this study are not applicable to the EDOU set-up as the timing of TTS was 48 hrs.
Howard et al., 2006 [23]	Study at a community level-II trauma center with fewer trauma management facilities emphasizes the significance of TTS in trauma patients. Results in terms of number of patients as well as number of injuries. Standardized TTS form. TTS within 24 hrs. Partial blinding. Low risk of bias.	Single-center. No use of power calculations. Small population sample.	TTS performed at 24 hours increased the rate of missed injury detection & can be implemented in the EDOU.
Biffl et al., 2003 [24]	Standardized TS documentation. Established timing of TS at 24 hrs. Comprehensive data from trauma registry. Use of power calculations.	Unconventional definition of missed injuries. Moderate risk of bias.	TTS performed at 24 hours increased the rate of missed injury detection & can be implemented in the EDOU.
Buduhan et al., 2000 [25]	Good study to identify the need for re-assessment to reduce the number of missed injuries, encouraging the need for further re-assessment of patients.	TTS is a surrogate measure. TTS is not assessed or utilized in the study. Moderate risk of bias.	The results of this study are irrelevant to the EDOU cohort, as TTS is a surrogate measure.
Janjua et al., 1998 [26]	TTS performed by a single resident physician (consistency). Clearly defined types of missed injuries. TTS within 24 hours	TTS performed by a single resident physician (bias). Advancements in trauma management tactics, development of dedicated trauma teams, and increased availability of advanced medical investigative technology in the last 18 years since this study may have an impact on the number of injuries missed at primary and secondary surveys. Moderate risk of bias.	The results of this study emphasize the need for TTS at 24 hours after admission and are quite relevant to major trauma patients admitted to the EDOU.
Enderson et al., 1990 [27]	Trauma registry consulted to include maximum number of patients. Detailed documentation of findings of primary, secondary & tertiary survey. Low risk of bias.	Primary focus is on patients with critically ill major trauma patients.	Only critically ill patients are included in this study. Timing of TS is not defined and is not applicable to the EDOU setup.

Primary Outcome

**Figure 2:**
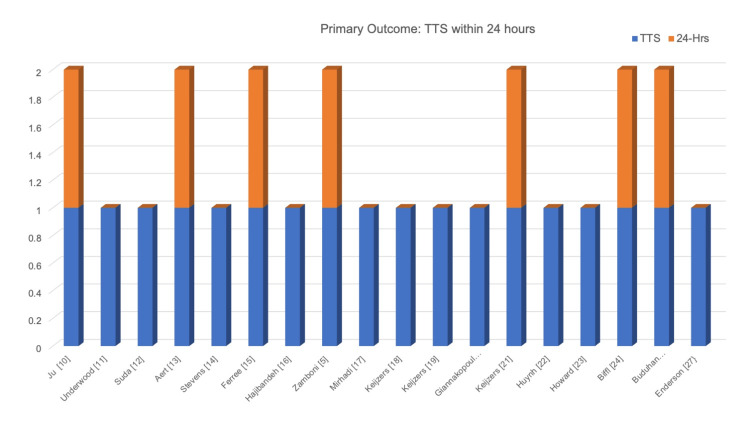
Primary outcome - tertiary trauma survey within 24 hours

**Figure 3:**
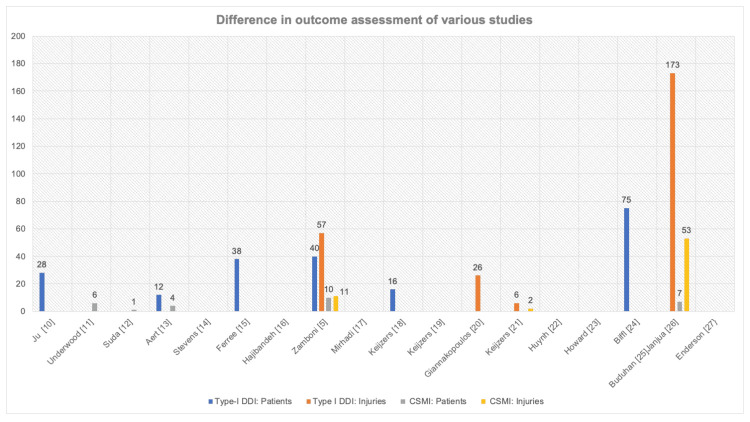
Difference in outcome assessment of various studies Type-I DDI: patients - number of patients with type-I delayed diagnosis of injury; Type-I DDI: injuries - number of type-I delayed diagnosis of injury; CSMI patients - number of patients with clinically significant missed injuries; CSMI injuries - number of clinically significant missed injuries DDI - delayed diagnosis of injury

This systematic review showed that one of the studies was conducted in the EDOU setting. However, the population in a study by Aert et al., 2020 [13] is closest to the ED population as it consists of patients with mild to moderate trauma, and all the patients underwent TTS at 24 hours. In this study, the detection rate of type-I DDIs was 3.1% (n=12), of which four patients had clinically significant missed injuries (CSMI). TTS was strictly carried out at 24 hours in seven (47%) of the studies on 10399 patients, Figure 2 [5,10, 13, 15, 21, 24, 26]. Five of the studies reported type-I DDI in terms of the number of patients, with a detection rate of 1.9% in 10193 patients [5,10, 13, 15, 24], while one reported type-I DDIs as 173 injuries at a detection rate of 56% [26]. Due to heterogeneity in methodology and outcome assessment, comprehensive statistical analysis is not possible (Figure 3).

Secondary Outcomes

**Figure 4:**
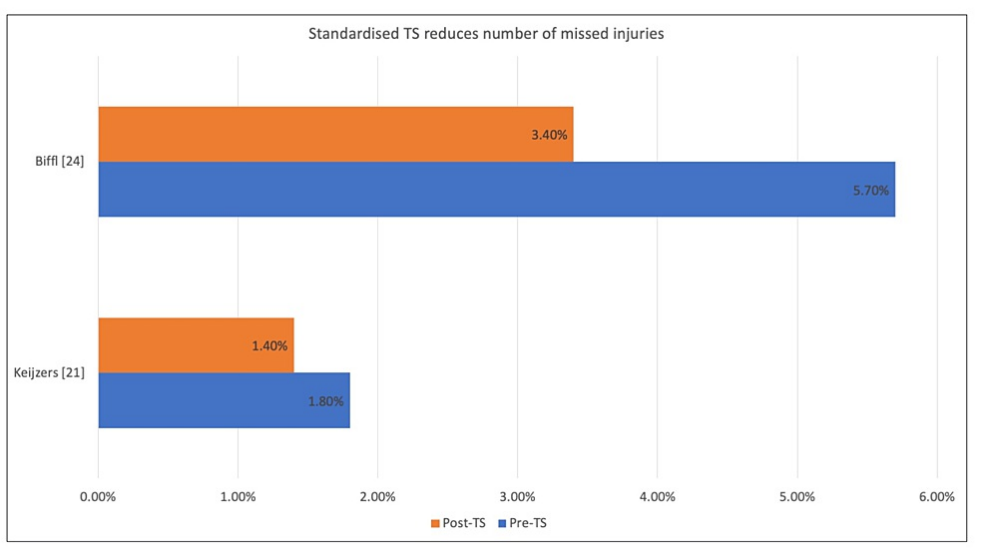
Impact of standardized tertiary survey on missed injury rate TS - tertiary survey

Despite poor compliance towards the use of structured TTS documentation, two studies (Figure 4) showed a reduction in the number of missed injuries upon the introduction of a standardized TS [21, 24]. In a study done by Enderson et al., 1990, the missed injury detection rate increased from 2% to 9% after introducing standardized TS [27]. These findings were echoed in a later study by Biffl et al. 2003, demonstrating a 36% reduction in type-I missed injuries after implementing standardized TS.

Most of the studies were conducted on ICU patients or those patients with significant and life-threatening injuries. Due to the severity of initial injuries, prioritization of management, and distraction, the number of missed injuries detected at TS in these patients is much higher [10, 12, 15, 22, 24, 27]. Although this result is not relevant to the EDOU cohort, it emphasizes the importance of TTS in all polytrauma patients. Another important observation from this literature review is that special skills or advanced training are not a prerequisite to conduct TTS. In different studies, TTS was carried out by a variety of physicians, including senior registrar [11], trainee physicians [5], surgical residents [20], or dedicated research assistants [21] with various levels of training. These findings were substantiated by Huynh et al. 2010, where the mid-level providers (MLP) were assigned to carry out TTS with satisfaction [22]. This study also showed appropriate assessment and a reduction of workload on trainee residents by 1802 hours.

The rate of DDIs was much higher at 56% type-I injuries, and 90% of clinically significant injuries were detected within 24 hours in a previous study by Janjua et al., 1998 [26]. However, this study only included patients admitted to intensive care units, missing out on patients with mild-to-moderate trauma. This high number could be due to a difference in the injury patterns and initial management priorities. Another more recent retrospective analysis by Ju et al., 2022, shows a significantly high rate of DDIs, 23.5% on a 24-hour TTS in patients with severe trauma [10]. The lower rate of DDIs in comparison to the earlier study by Janjua et al., 1998 could be due to the advancements in trauma management over the last two decades. While this patient cohort is not representative of the EDOU population, it reiterates the importance of TTS at 24 hours. Furthermore, the findings of the study by Janjua et al., 1998 could well be representative of the LMICs and healthcare systems with less-developed trauma services.

Discussion

There is an obvious paucity of literature regarding TTS and a definite scarcity regarding its role in the setting of emergency OM. The single most important and relevant study is a retrospective cohort study by van Aert et al., 2020, conducted in the Netherlands [13]. This study was conducted at a level-II TC, which would be the closest representation of an LMIC in the existing literature. Furthermore, the study group was confined to those with mild to moderate trauma on initial assessment and no obvious serious injuries. TTS was time-defined at 24 hours. These characteristics are closest to the cohort who would be admitted to EDOU. The outcome of this study is described in terms of number of injuries rather than number of patients, making it more substantial. The reported DDI rate was 3.1%, and the rate of prevention of serious damage was 0.5%. These results are consistent with results from several other level-II TCs [11, 18, 21-23]. These figures may appear insignificant as the initial quality of primary and secondary surveys is possibly much more advanced and not comparable to those of LMICs or under-resourced trauma services. However, in alternate settings, the yield of TTS in EDOU is likely higher and more significant. Research needs to be conducted in LMICs to support this theory. Overall, the findings of this study demonstrate the need to explore the role of TTS on EDOU in LMICs. Furthermore, as some LMICs might not have EOUs, the results of this review and further studies could encourage the development of EDOUs in LMICs.

Similar results were also seen in an earlier study by Keijzers et al., 2011, where TTS was performed in a regional TC without a dedicated trauma service [21]. The DDI rate was 3.2% in patients undergoing TTS at 24 hours. While the inclusion criteria of the patients are closest to that of the EDOU cohort, and the lack of dedicated trauma services makes the settings of this study somewhat comparable to the LMICs, the compliance rate of TTS was only 20%, leaving room for interpretation.

Most studies also concluded satisfactorily that standardized TTS at 24 hours gives better outcomes and is a cost-effective, easy, and efficient tool [10-12, 22-24, 27].

In a nutshell, it is evident from this systematic literature review that regardless of the clinical setup and severity of the injury, a standardized tertiary reassessment at 24 hours can identify a significant number of DDIs, and it does not require any additional resources or advanced training.

Limitations

This systematic literature review is limited by the absence of dedicated studies from EDOU and a deficit in the overall trauma statistics from developing and LMIC.

## Conclusions

There is an obvious knowledge gap about the import of TTS in OM. However, current evidence does emphasize the significance of TTS in identifying injuries missed on the initial primary and secondary survey. There is a consensus that standardized TTS within 24 hours reduces the rate of DDI in poly-trauma patients, regardless of the severity of injury and the clinical setting where TTS is performed. This indicates a promising role for TTS in improving the overall standard of trauma care and reducing its resultant socio-economic burden on society. By and large, the introduction of a simple standardized TTS proforma could take trauma management to the next level, especially in LMICs and in resource-poor settings with underdeveloped trauma services. It is a simple, cost-effective tool that does not require advanced training or exceptional skill and can be carried out by less experienced physicians and MLPs. There is sufficient information that the integration of TTS with OM will likely reduce the number of DDIs unnecessary hospital admissions and act as a safety netting mechanism for poly-trauma patients with mild-to-moderate injuries who did undergo initial WBCT. Large-scale, multi-center, preferably RCTs, especially from LMICs with limited trauma services and networking, are recommended to validate the conclusions of this systematic literature review.
